# Spatial transcriptomics maps early B-cell and T-cell activation in hidradenitis suppurativa

**DOI:** 10.1016/j.jid.2026.01.041

**Published:** 2026-02-19

**Authors:** Jongeun Lee, Seoyoon Ham, Jongmi Lee, James G. Krueger, Young In Lee, Jaehwan Kim

**Affiliations:** 1 Laboratory for Investigative Dermatology, The Rockefeller University, New York, New York, USA;; 2 Department of Dermatology, Cutaneous Biology Research Institute, Yonsei University College of Medicine, Seoul, Republic of Korea;; 3 Department of Dermatology, University of California, Davis, Sacramento, California, USA;; 4 Dermatology Section, Veterans Affairs Northern California Health Care System, Mather, California, USA;; 5 Scar Laser and Plastic Surgery Center, Yonsei Cancer Hospital, Seoul, Republic of Korea; 6 These authors contributed equally as co-first authors.; 7 These authors are co-corresponding authors.

**Keywords:** B-cells, Hidradenitis suppurativa, Spatial transcriptomics, Single-cell RNA sequencing, Type 17 T cells

## Abstract

Hidradenitis suppurativa is a chronic, debilitating inflammatory skin disease that progresses from superficial nodules in early stages to dermal tunnels, tertiary lymphoid structures, and fibrosis in advanced lesions. Interplays between dermal tunnel keratinocytes, tertiary lymphoid structures, and fibroblasts promote pathogenic T-cell and B-cell inflammation in late-stage disease. However, whether these immune activations sequentially precede or follow late-stage structure formation is unclear. To delineate the temporal sequence of immune activation, we integrated spatial and single-cell transcriptomic analyses of early- and late-stage hidradenitis suppurativa and compared them with those of acne conglobata and psoriasis. Spatial transcriptomics of 156 regions of interest revealed robust dermal immune activation in early-stage hidradenitis suppurativa, including B-cell enrichment, type 17 T-cell pathway activation, and neutrophilic infiltration, occurring prior to tunnel or tertiary lymphoid structure formation. Single-cell RNA sequencing confirmed early plasma cell expansion and identified multifunctional B cells expressing proinflammatory cytokines, antigen-presentation machinery, and Igs. Notably, T cells rather than fibroblasts were the predominant producers of the B-cell chemoattractant *CXCL13* in early lesions. Together, these findings demonstrate that hidradenitis suppurativa exhibits early and sustained immune activation that precedes dermal remodeling, underscoring the aggressive nature from its onset and suggesting that early therapeutic targeting of the type 17 T-cell axis and B-cell–mediated inflammation may help prevent disease progression.

## INTRODUCTION

Hidradenitis suppurativa (HS) is a debilitating chronic inflammatory skin disorder that significantly impacts the patient’s QOL and poses therapeutic challenges (Jemec, 2012). HS follows a progressive course, with early-stage HS (early HS) presenting as inflammatory nodules and abscesses predominantly affecting intertriginous regions such as the axillae, groin, and inframammary areas. This eventually leads to severe tissue damage, forming dermal tunnels, extensive fibrosis, and scarring in later stages (late HS) (Sabat et al, 2025).

Although follicular occlusion has long been considered a primary driver of HS, emerging evidence suggests a more complex immunopathological process involving innate and adaptive immune responses (Jastrząb et al, 2023; Krueger et al, 2024; Shishido-Takahashi et al, 2025; Yu et al, 2024). Growing evidence indicates that HS pathogenesis involves B-cell activation, dysregulation of type 17 T (T17) cell pathway, and neutrophilic activity (Frew, 2025; Gudjonsson et al, 2020; Lowe et al, 2020; Malvaso et al, 2023; Sabat et al, 2023). A recent study demonstrated that in advanced lesions, B cells and plasma cells aggregate around epithelialized tracts, forming tertiary lymphoid structures (TLSs), alongside pathogenic T17 cell, regulatory T cells, and follicular helper T cells (Lowe et al, 2024; Yu et al, 2024). In addition, elevated autoantibody levels have been identified in HS patient sera, particularly in those with Hurley stages II–III as well as within stage III HS skin lesions compared with healthy controls (Carmona-Rivera et al, 2022).

Despite increasing recognition of immune dysregulation underlying HS, much of the prior research has focused on later disease stages, where irreversible tissue damage has already occurred. As a result, there is an unmet need and incomplete immunologic understanding of early-stage disease, highlighting a critical gap in our knowledge of early disease mechanisms. Moreover, no systemic therapeutics are currently available for the early-stage HS, and none of the studies have specifically targeted early lesions with the aim of preventing disease progression. Investigating early-stage inflammation is therefore crucial to identify potential therapeutic interventions that could halt progression to severe, treatment-resistant HS.

In this study, we employed spatial and single-cell transcriptomics to comprehensively characterize early and late HS, comparing them with acne conglobata, which shares follicular occlusion as a common pathophysiological feature with HS (Jastrząb et al, 2023). Throughout the manuscript, we use the term T17 cell to broadly refer to IL-17–producing T cells, encompassing both CD4^+^ and CD8^+^ subsets, to capture the full spectrum of IL-17–associated T-cell responses observed in HS lesions. Our findings reveal that HS skin exhibits extensive dermal immune activation even in the early stage before late-stage structures (dermal tunnels, TLSs, and fibrosis) are formed, with B cells and IL-17–driven inflammation playing a dominant role. Notably, deep dermal inflammation was consistently observed in the early stage of HS skin, suggesting that although follicular occlusion may contribute to disease initiation, additional inflammatory mechanisms drive pathology beyond the hair follicle.

## RESULTS

### Early HS without dermal tunnels, TLSs, and fibrosis is still enriched with transcripts associated with B cells, cytotoxic T cells, T17 cell pathway, and neutrophils

Histological analysis of early HS showed widening of hair follicles with neutrophilic and lymphocytic infiltrates in the superficial dermis and around hair follicles (Supplementary Figure S1a). The inflammatory infiltrates were also present in the deep dermis, independent of hair follicles. In contrast, late HS was characterized by epithelialized dermal tunnels in the deep dermis with nearby immune cell aggregates (Supplementary Figure S1b). CD3 and CD20 staining of late HS tissues revealed that these aggregates were primarily composed of T cells and B cells, consistent with the recently described TLSs in HS (Lowe et al, 2024; Yu et al, 2024) (Supplementary Figure S2).

**Figure 1.**
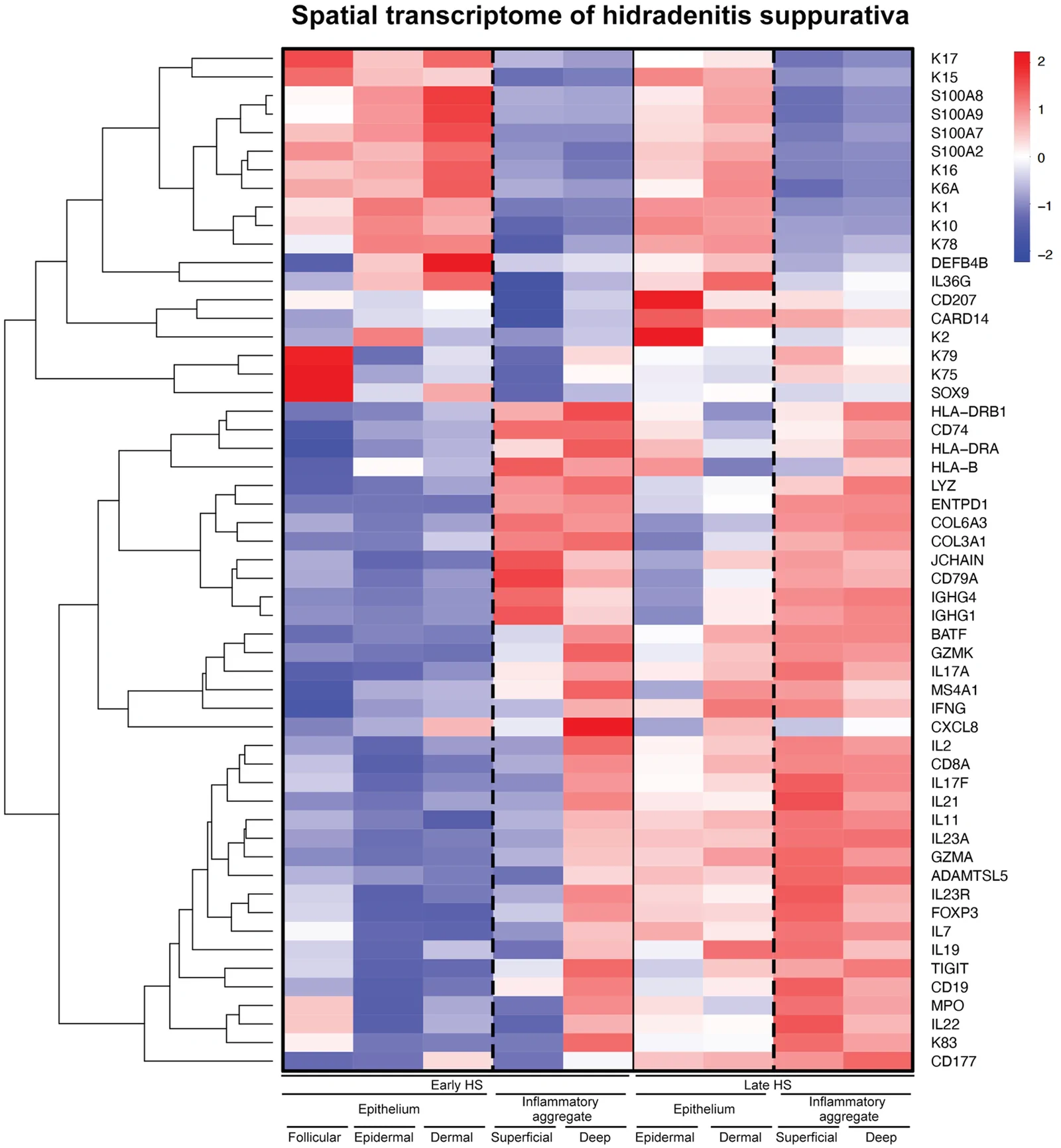
Spatial transcriptome profile of HS. Shown is a heatmap depicting different average gene expression across ROIs in early- and late-stage HS. HS, hidradenitis suppurativa; ROI, region of interest.

To overview spatially different inflammatory microenvironments of early and late HS, we compared the average gene expression in follicular, epidermal, and dermal epithelium as well as in superficial and deep dermal inflammatory aggregates from early and late HS (Figure 1). In early HS, dermal epithelium corresponded to the follicular cyst wall and epidermal appendages within the dermis, whereas in late HS, it represented the wall of dermal tunnels.

The epidermal epithelium in both early and late HS exhibited high expression of antimicrobial peptide (AMP) transcripts, including *S100A2, S100A7, S100A8, S100A9*, and *DEFB4B* (Abdi et al, 2024; Kim et al, 2023a; Liang et al, 2023; Saito-Sasaki and Sawada, 2023) (Figure 1). In addition, T17 cell response–enhancing cytokine (*IL36G* [Elias et al, 2021]) and stress keratins (Cohen et al, 2024) (*K6A*, *K16*, and *K17*) were highly expressed in the epidermal epithelium, with stronger expression observed in the dermal epithelium of both early and late HS.

In early HS, superficial inflammatory aggregates were enriched with transcripts associated with antigen presentation, including major histocompatibility complex (MHC) class I molecule (*HLA-B*), MHC class II molecules (*HLA-DRB1* and *HLA-DRA*), and MHC class II–trafficking regulator *CD74* (Schröder, 2016). In addition, B-cell lineage markers (Adams et al, 2009) (*CD19*, *MS4A1* [CD20], and *CD79A*) along with Ig transcripts (*IGHG1* and *IGHG4*) were prevalent. Notably, deep inflammatory aggregates in early HS exhibited high expression of cytotoxic T-cell transcripts (Bratke et al, 2005; Kim et al, 2021) (*CD8A*, *GZMA*, and *GZMK*), T17 cell–activating cytokine (*IL23A* [Liu et al, 2020]), and T17 cell cytokines (Kim et al, 2025, 2021; Lee and Kuchroo, 2015) (*IL17A*, *IL17F*, *IL22*, *IL21*, and *IFNG*). Neutrophil-enriched transcripts (*MPO* [Lin et al, 2024] and *CD177* [Stroncek et al, 2004]) and a potent neutrophil chemoattractant (*CXCL8* [Metzemaekers et al, 2020]) were also highly expressed. In late HS, transcripts enriched in early HS inflammatory aggregates, including those associated with B cells, cytotoxic T cells, T17 cell pathway, and neutrophils, remained highly expressed in both superficial and deep inflammatory aggregates.

These findings indicate that early HS, without late HS structures such as dermal tunnels, TLSs, and fibrosis, is enriched with transcripts associated with B cells, cytotoxic T cells, T17 cell pathway, and neutrophils.

### Early HS inflammatory microenvironments differ between the superficial and deep dermis

**Figure 2.**
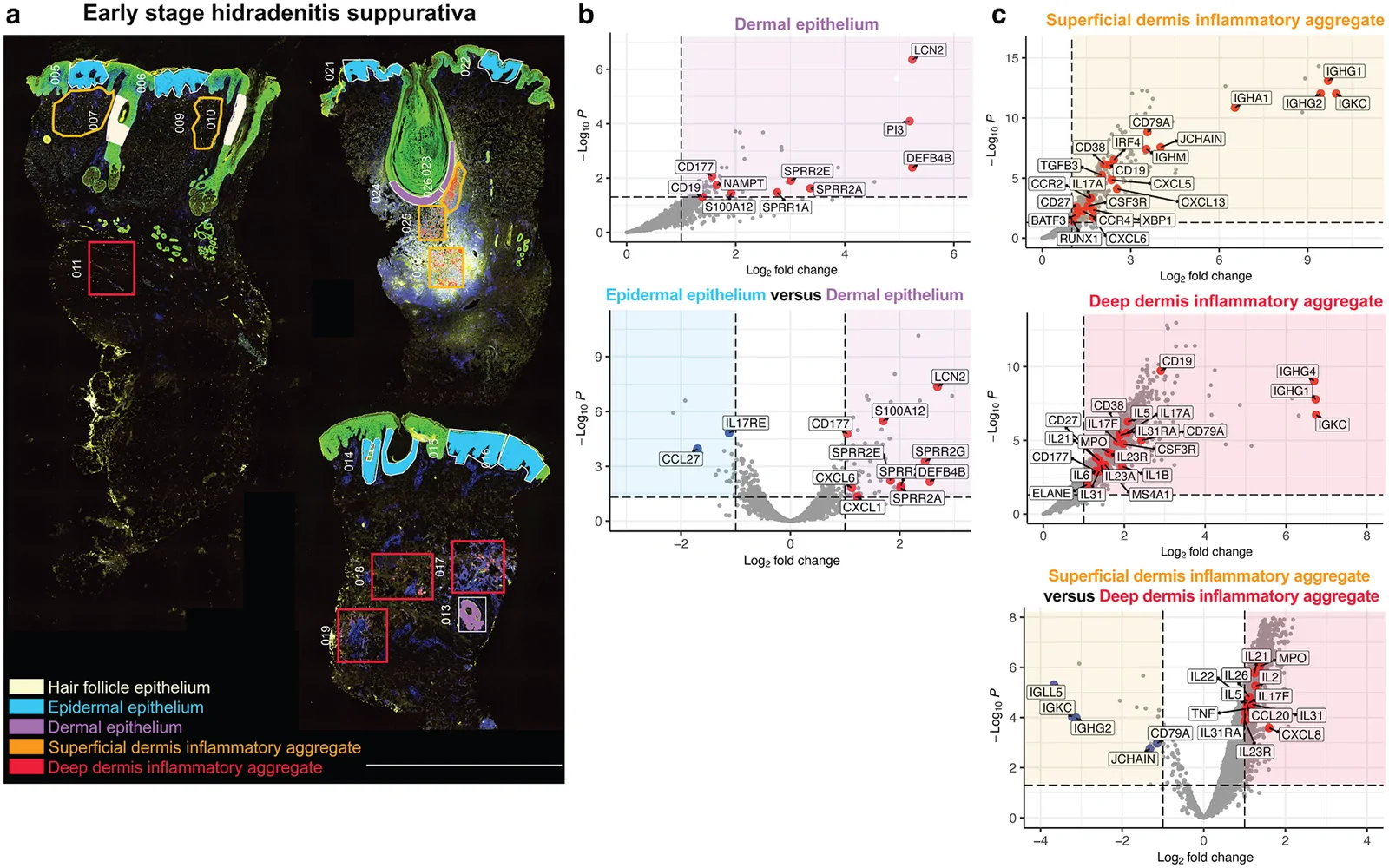
Spatial transcriptomic profile of early-stage HS. **(a)** Representative image illustrating the selection of ROIs in the early-stage HS biopsy tissue sample. Bar = 2.5 mm. **(b)** Volcano plots displaying DEGs in the dermal epithelium of early-stage HS compared with that of epidermal epithelium of acne conglobata (control) (upper panel) and between the epidermal and dermal epithelium of early-stage HS (lower panel). **(c)** Volcano plots displaying DEGs in the superficial dermis inflammatory aggregate of early-stage HS compared with that of the control (upper panel), in deep dermis inflammatory aggregate of early-stage HS compared with that of control (middle panel), and between superficial and deep dermal inflammatory aggregates of early-stage HS (lower panel). DEG, differentially expressed gene; HS, hidradenitis suppurativa; ROI, region of interest.

To investigate spatial variation in early HS, we analyzed differentially expressed genes in epidermal and dermal epithelium as well as in superficial and deep dermal inflammatory aggregates using acne conglobata epidermal epithelium as a control (Figure 2a).

In the early HS dermal epithelium, transcripts for AMPs (*S100A12* [Jackson et al, 2017], *DEFB4B* [Harder et al, 1997], and *PI3* [Baranger et al, 2008]), small proline-rich proteins (Zhang et al, 2022) (*SPRR1A*, *SPRR2A*, and *SPRR2E*), B-cell lineage marker (*CD19* [Adams et al, 2009]), and neutrophil-enriched genes (*CD177* [Stroncek et al, 2004], *NAMPT* [Siakaeva et al, 2019], and *LCN2* [Ma et al, 2022]) were upregulated compared with the control (fold change [FCH] > 2 and false discovery rate [FDR] < 0.05) (Figure 2b). When comparing epidermal with dermal epithelium in early HS, the expressions of the AMP transcripts, neutrophil marker (*CD177* [Stroncek et al, 2004]), and neutrophil chemoattractants (Metzemaekers et al, 2020) (*CXCL1* and *CXCL6*) were higher in dermal epithelium (FCH > 2 and FDR < 0.05) (Figure 2b).

Both superficial and deep inflammatory aggregates in early HS demonstrated markedly increased expression of B-cell lineage markers (Adams et al, 2009) (*CD19* and *CD79A*), plasma cell surface markers (Brynjolfsson et al, 2018; Kassambara et al, 2015) (*CD38* and *CD27*), Ig (*IGHG1*), T17 cell cytokine (Lee and Kuchroo, 2015) (*IL17A*), and neutrophil surface molecule (*CSF3R* [Pazhakh et al, 2017]) (FCH > 2 and FDR < 0.05) (Figure 2c). When superficial and deep inflammatory aggregates were compared in early HS, B-cell transcript (*CD79A* [Adams et al, 2009]), plasma cell transcript (*JCHAIN* [Xu et al, 2020]), and Ig genes were more expressed in the superficial dermis than in the deep dermis (FCH > 2 and FDR < 0.05) (Figure 2c). In contrast, T17 cell cytokines (Liu et al, 2020) (*IL17F*, *IL21*, *IL22*, *IL23R*, *IL26*, and *TNF*), T17 cell–recruiting chemokine (*CCL20* [Harper et al, 2009]), and neutrophil-enriched transcript (*MPO* [Lin et al, 2024]) genes were more expressed in the deep dermis than in the superficial dermis (FCH > 2 and FDR < 0.05).

These findings indicate that early HS inflammatory microenvironments are different between the superficial and deep dermis. B cells are more activated in the superficial dermis, whereas T17 cells and neutrophils are more activated in the deep dermis.

### Late HS reveals full-thickness inflammatory microenvironments

**Figure 3.**
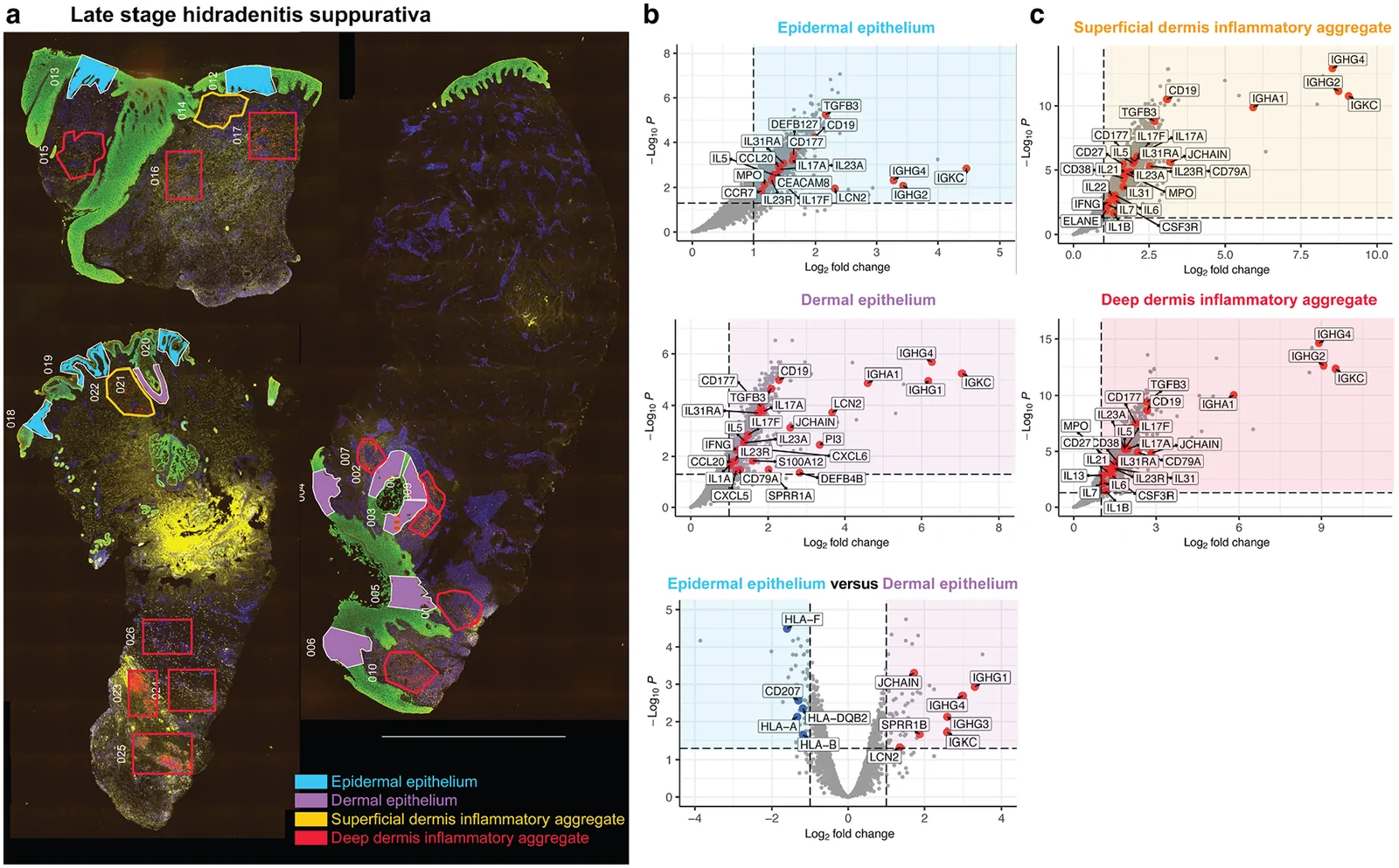
Spatial transcriptomic profile of late-stage HS. **(a)** Representative image illustrating the selection of ROIs in the late-stage HS biopsy tissue sample. Bar = 2.5 mm. **(b)** Volcano plots displaying DEGs in the epidermal epithelium of late-stage HS compared with that in epidermal epithelium of acne conglobata (control) (upper panel), in the dermal epithelium of late-stage HS compared with that in the control (middle panel), and between epidermal and dermal epithelium of late-stage HS (lower panel). **(c)** Volcano plot displaying DEGs in the superficial dermis inflammatory aggregates of late-stage HS compared with that of the control (upper panel) and in the deep dermis inflammatory aggregates of late-stage HS compared with that of the control (lower panel). DEG, differentially expressed gene; HS, hidradenitis suppurativa; ROI, region of interest.

As in the early HS, we investigated spatially different inflammatory microenvironments in late HS by differential expression testing (Figure 3a). Both epidermal and dermal epithelium in late HS demonstrated increased expression of B-cell marker (*CD19* [Adams et al, 2009]), Igs, T17 cell–recruiting chemokine (*CCL20* [Harper et al, 2009]), T17 cell–inducing transcript (*TGFB3* [Duesman et al, 2023; Liu et al, 2015; Sharma et al, 2013]), T17 cell–activating cytokine (*IL23A* [Liu et al, 2020]), T17 cell cytokines (Lee and Kuchroo, 2015; Liu et al, 2020) (*IL17A*, *IL17F*, *IL21*, *IL23R*, and *TGFB3*), and neutrophil-enriched transcripts (*CD177* [Stroncek et al, 2004] and *LCN2* [Ma et al, 2022]) (FCH > 2 and FDR < 0.05) (Figure 3b). When epidermal and dermal epithelium were compared, the expressions of Langerhans cell marker (*CD207* [Hunger et al, 2004; Romani et al, 2010]) and MHC classes I and II molecules were higher in the epidermal epithelium (FCH > 2 and FDR < 0.05) (Figure 3b). In contrast, plasma cell transcript (*JCHAIN* [Xu et al, 2020]), Ig genes, and neutrophil-enriched transcript (*LCN2* [Ma et al, 2022]) were more highly expressed in the dermal epithelium (FCH > 2 and FDR < 0.05).

In late HS, both superficial and deep dermis inflammatory aggregates demonstrated a pronounced upregulation of B-cell markers (Adams et al, 2009) (*CD19* and *CD79A*), plasma cell–related transcripts (*JCHAIN* [Xu et al, 2020], *CD38*^29^, and *CD27*^30^), Igs, T17 cell–inducing transcripts (Liu et al, 2020) (*TGFB3* [Duesman et al, 2023; Liu et al, 2015; Sharma et al, 2013], *IL1B* [Liu et al, 2020], and *IL6* [Liu et al, 2020]), T17 cell–activating cytokine (*IL23A* [Liu et al, 2020]), T17 cell cytokines (Lee and Kuchroo, 2015) and receptor (*IL17A*, *IL17F*, *IL21*, *IL23R*), and neutrophil-enriched transcripts (*MPO* [Lin et al, 2024], *CD177* [Stroncek et al, 2004], and *CSF3R* [Pazhakh et al, 2017]) compared with the control (FCH > 2 and FDR < 0.05) (Figure 3c). In contrast to early HS, comparison of superficial with deep dermal inflammatory aggregates in late HS did not reveal substantial differences in gene expression profiles, indicating broadly similar inflammatory signatures across the full dermal thickness.

Together, these findings indicate that late HS inflammatory microenvironments are characterized by widespread B-cell, T17 cell, and neutrophil activation in both superficial and deep dermis. Dermal tunnels in the deep dermis interacting with B cells and neutrophils may contribute to the evolving full-thickness inflammation in late HS.

### HS inflammatory microenvironments are established in the deep dermis from the early stage of the disease

**Figure 4.**
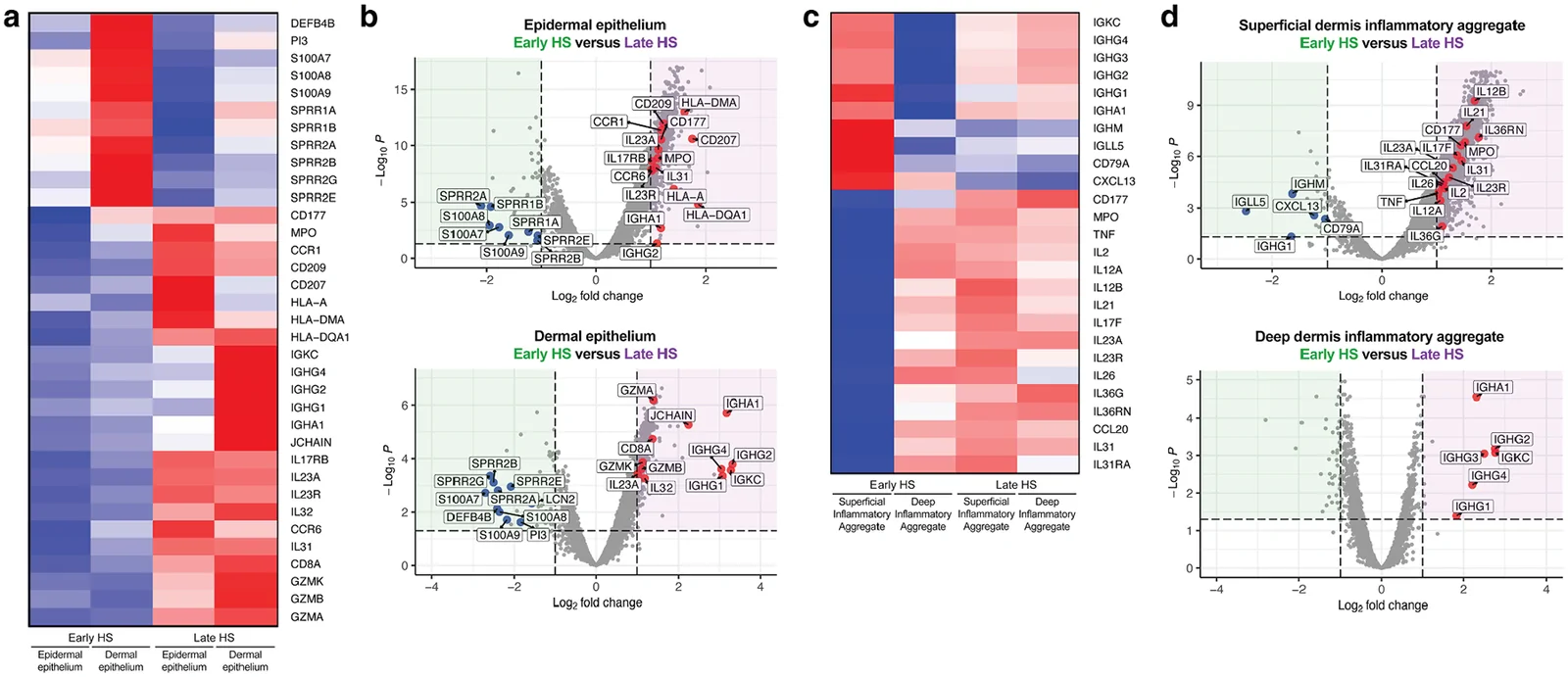
Comparative spatial transcriptomic analysis of early- and late-stage HS. **(a)** Heatmap illustrating differential average gene expression across the epidermal and dermal epithelium in early- and late-stage HS. **(b)** Volcano plots showing DEGs between the epidermal epithelium of early- and late-stage HS (upper panel) and between the dermal epithelium of early- and late-stage HS (lower panel). **(c)** Heatmap illustrating differential average gene expression across superficial and deep dermal inflammatory aggregates in early- and late-stage HS. **(d)** Volcano plots displaying DEGs between superficial dermal inflammatory aggregates of early- and late-stage HS (upper panel) and between deep dermal inflammatory aggregates of early- and late-stage HS (lower panel). DEG, differentially expressed gene; HS, hidradenitis suppurativa.

To gain insights into the evolving inflammatory microenvironments in HS, we compared corresponding regions of early and late HS (Figure 4). Differential expression testing demonstrated progressive increases from early to late HS, including (i) Langerhans cell marker (*CD207* [Hunger et al, 2004; Romani et al, 2010]), antigen-presenting cells (APCs) marker (*CD209* [Engering et al, 2002; Geijtenbeek et al, 2000]), and MHC classes I and II molecules in the epidermal epithelium; (ii) plasma cell–related transcript (*JCHAIN* [Xu et al, 2020]) in the dermal epithelium; (iii) Igs in the epidermal and dermal epithelium and deep dermis inflammatory aggregates; (iv) cytotoxic T-cell transcripts (Bratke et al, 2005; Kim et al, 2021) (*CD8A*, *GZMA*, *GZMB*, and *GZMK*) in the dermal epithelium; (v) T17 cell–activating cytokine (*IL23A* [Liu et al, 2020]) in epidermal and dermal epithelium and superficial dermis inflammatory aggregates; (vi) T17 cell–recruiting chemokine (*CCL20* [Harper et al, 2009]), T17 cell cytokines (Liu et al, 2020) and receptor (*IL17F*, *IL21*, *IL26*, *IL23R*, and *TNF*), and T17 cell response–enhancing cytokine (*IL36G* [Elias et al, 2021]) in superficial dermis inflammatory aggregates; (vii) neutrophil-enriched transcripts (*MPO* [Lin et al, 2024], *CD177* [Stroncek et al, 2004], and *LCN2* [Ma et al, 2022]) in epidermal and dermal epithelium and superficial dermis inflammatory aggregates; and (viii) regulatory cytokines (*IL2* [Kim et al, 2017; Kryczek et al, 2007] and *IL36RN* [Queen et al, 2019]) in superficial dermis inflammatory aggregates (FCH > 2 and FDR < 0.05).

In contrast, early HS exhibited higher expressions than late HS of (i) small proline-rich proteins (Zhang et al, 2022) and S100 proteins (Abdi et al, 2024; Liang et al, 2023; Saito-Sasaki and Sawada, 2023) in the epidermal and dermal epithelium and (ii) B-cell chemoattractant (*CXCL13* [Takagi et al, 2008]), B-cell marker (Adams et al, 2009) (*CD79A*), and Igs in superficial dermis inflammatory aggregates (FCH < 0.5 and FDR < 0.05).

In the deep dermis inflammatory aggregates, only Igs were differentially expressed between early and late HS, reinforcing the idea that the deep dermis is already highly inflamed in early disease (Figure 4c and d).

Taken together, these findings underscore active B-cell engagement and presence of deep inflammation from the early stage of the disease. With disease progression, antigen presentation expands in the epidermis, and regulatory immune mechanisms emerge in the dermis.

### Single-cell transcriptomics validate spatial transcriptomic findings underscoring the role of B cells beyond antigen presentation and antibody production

**Figure 5.**
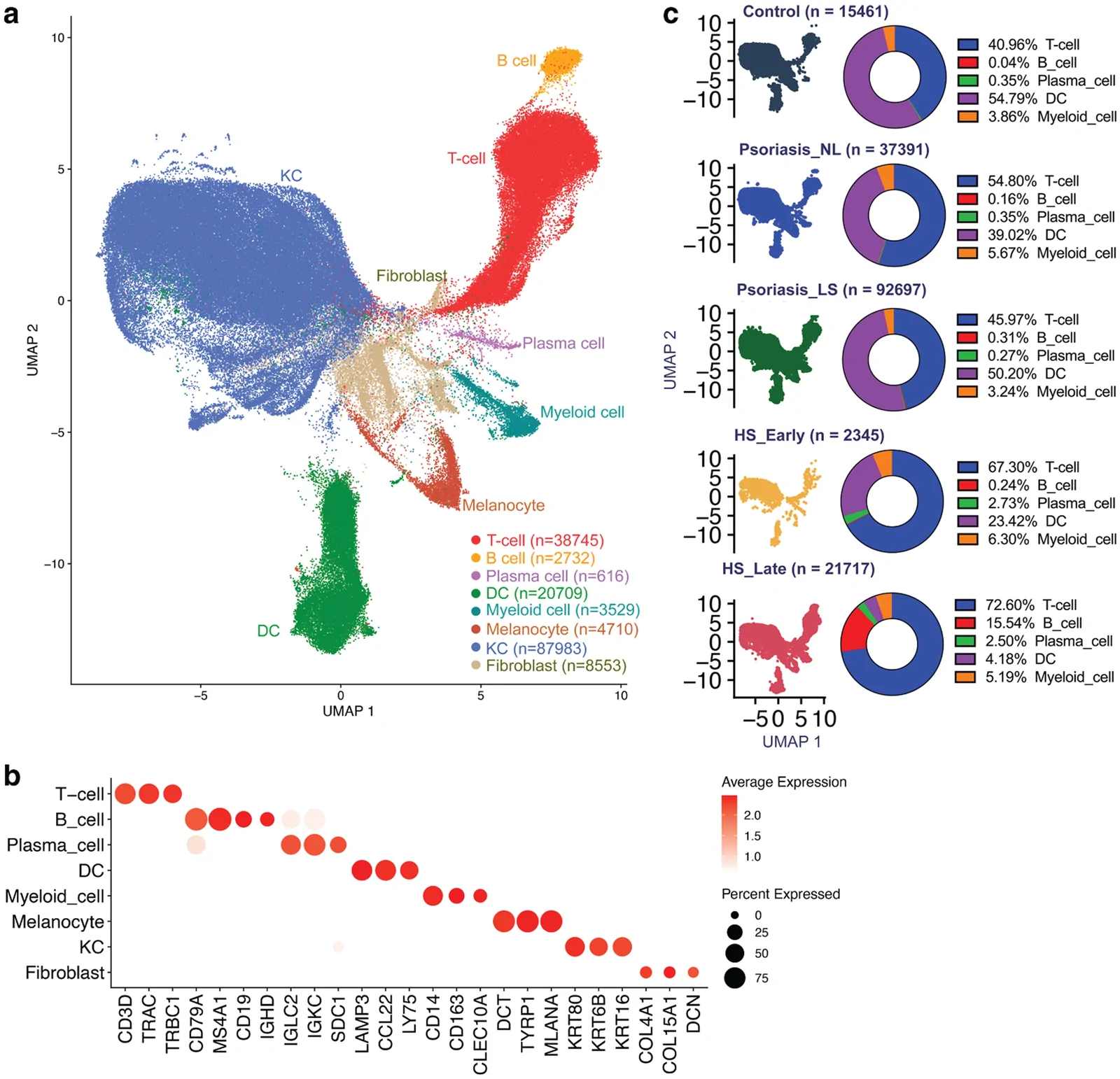
Single-cell transcriptomic characterization of immune and epithelial cell populations. **(a)** The UMAP plot showing all captured cell clusters across conditions. **(b)** Dot plot illustrating the expression levels of cluster-defining genes. **(c)** Relative proportions of T-cell, B-cell, plasma cell, DC, and myeloid cell clusters in control, psoriasis nonlesional and lesional skin, early-stage HS, and late-stage HS. Proportions shown in **c** represent relative frequencies within the immune cell compartment only. Keratinocytes and fibroblasts are included in the UMAP for contextual reference but were excluded from proportion calculations owing to the known selection bias of the migrating (“crawled-out”) cell isolation method toward immune cells. DC, dendritic cell; HS, hidradenitis suppurativa; UMAP, Uniform Manifold Approximation and Projection.

To validate spatial transcriptomic findings, we studied single-cell transcriptomic profiles of early (n = 2345) and late (n = 21,717) HS with psoriasis lesional (n = 92,697) and nonlesional (n = 37,391) skin and control (n = 15,461) from a different cohort (Figure 5a). All single-cell libraries were generated by 1 investigator using the same experiment protocol, minimizing batch-to-batch variability (Kim et al, 2025, 2023a, 2023b, 2021). The average expression of canonical markers for each immune cell is shown in Figure 5b.

Cell-type proportions derived from single-cell data (Figure 5c) independently confirmed the B-cell enrichment in HS. Both early and late HS exhibited enrichment of plasma cells, comprising 2.50 and 2.73% of the total cell population, respectively, which is 7- to 10-fold higher than in control (0.35%) or nonlesional (0.35%) and/or lesional (0.27 %) psoriasis skin. Simultaneously, B-cell proportions surged dramatically from 0.24% in early HS to 15.54% in late HS.

**Figure 6.**
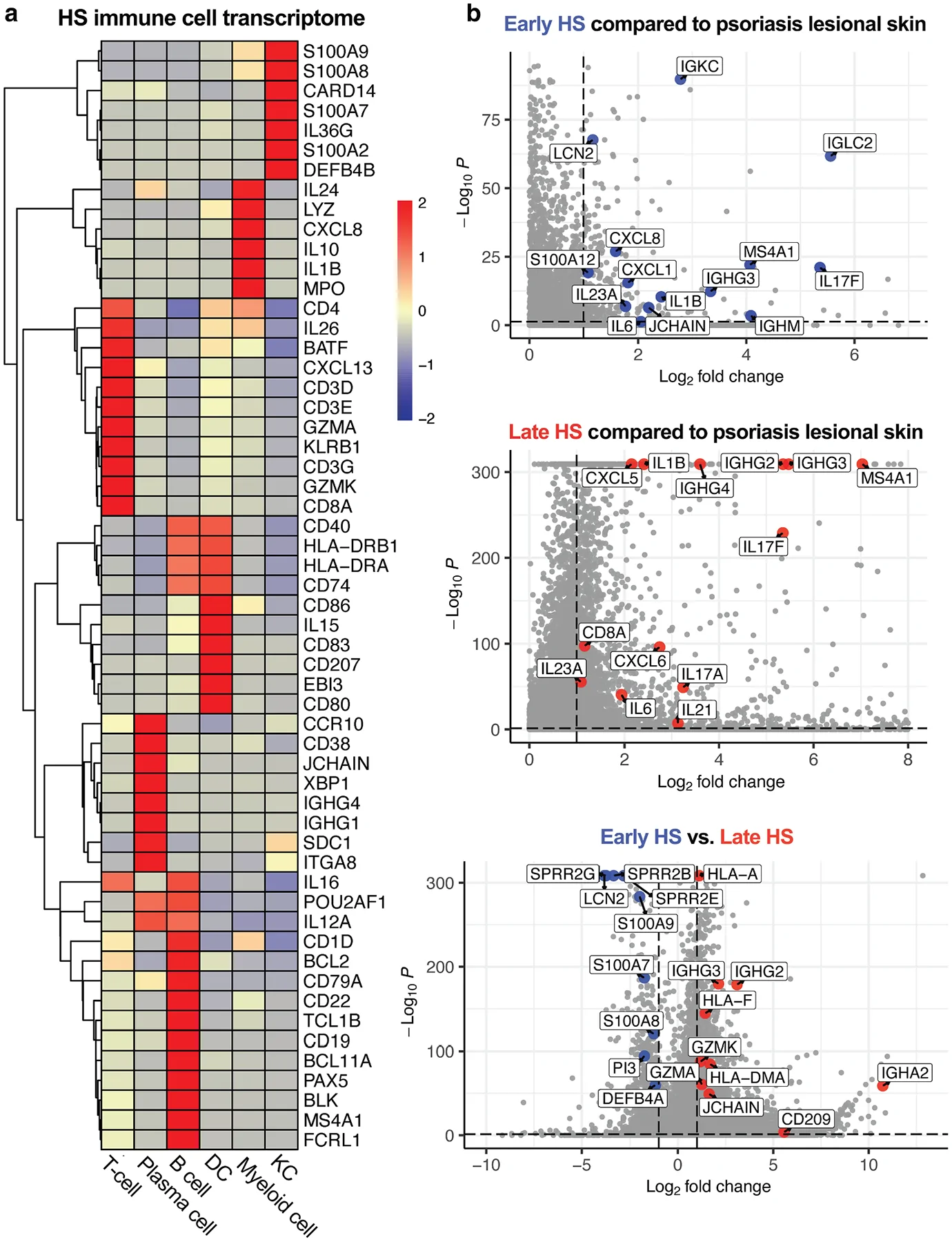
Single-cell transcriptomic profile of HS. **(a)** Heatmap illustrating differential average gene expression across various cell clusters, including T cells, plasma cells, B cells, DCs, myeloid cells, and keratinocytes. The T-cell heatmap represents the combined T-cell population, including both CD4^+^ and CD8^+^ T cells. Data are shown for the combined early- and late-stage HS single-cell dataset. **(b)** Volcano plots displaying DEGs between early-stage HS and psoriasis lesional skin (first panel), late-stage HS and psoriasis lesional skin (middle panel), and early- versus late-stage HS (last panel). DC, dendritic cell; DEG, differentially expressed gene; HS, hidradenitis suppurativa.

The average gene expression profiles of immune cells in HS were consistent with previous HS single-cell studies (Kim et al, 2023b) (Figure 6a): (i) T cells were enriched with cytotoxic T-cell transcripts (Bratke et al, 2005; Kim et al, 2021) (*CD8A*, *GZMA*, and *GZMK*) and T17 cell transcripts (*KLRB1* [Cosmi et al, 2008], *BATF* [Schraml et al, 2009; Yang et al, 2008], and *IL26* [Liu et al, 2020]); (ii) dendritic cells expressed core MHC class II antigen presentation machinery (*HLA-DRB1*, *HLA-DRA*, *CD74*), costimulatory molecules enhancing antigen presentation (*CD40*, *CD86*, *CD80*, *CD83*) (Shao et al, 2022), and Langerhans cell marker (*CD207* [Hunger et al, 2004; Romani et al, 2010]); and (iii) myeloid cells were the predominant sources of *IL1B* and *IL10*.

In addition, our single-cell analysis indicated that HS B cells could serve as APCs (Figure 6a), expressing antigen-presenting molecules (*CD40* [O’Sullivan and Thomas, 2003], *HLA-DRB1*, and *HLA-DRA*), along with B-cell markers (Adams et al, 2009) (*CD19*, *MS4A1* [CD20], and *CD79A*), B-cell receptor signaling molecule (*BLK* [Compeer et al, 2015]), transcription factors involved in B-cell development (*PAX5* [Calderón et al, 2021] and *BCL11A* [Yu et al, 2012]), and B-cell prosurvival protein (*BCL2* [Slomp and Peperzak, 2018]). Moreover, HS B cells may actively promote inflammation with high levels of *IL16* (Center and Cruikshank, 1982; Mathy et al, 2000), a chemoattractant for CD4^+^ cells and modulator of T-cell activation. Furthermore, HS T cells preferentially expressed B-cell chemoattractant *CXCL13* (Takagi et al, 2008), which was upregulated in the superficial dermis of early HS before fibrosis formation in the spatial transcriptomic data (Figures 2c and 4d).

To further explore the functional relevance of B-cell enrichment in HS, we investigated ligand–receptor interactions between immune cells in HS that are more elevated in HS than in psoriasis (Supplementary Figure S3). This revealed notable ligand–receptor interactions between B cells and plasma cells with other immune subsets, including *CXCR5*–*CXCL13* and *IL16*–*CD4* in HS (adjusted *P* < .05). These findings indicate that in contrast to psoriasis, B cells in HS are not only enriched but also actively participate in inflammatory networks with other immune cell subsets, reinforcing their pathogenic role.

To validate differentially expressed genes identified in the spatial transcriptomic data, we studied differentially expressed genes of early and late HS in the single-cell transcriptomic data, comparing them with psoriasis lesional skin, which shares T17 cell activation as a common pathophysiological feature with HS (Kim et al, 2023b) (Figure 6b). Consistent with spatial findings, both early and late HS demonstrated increased expression of B-cell lineage marker (Adams et al, 2009) (*MS4A1* [CD20]) and Igs, together with T17 cell–inducing transcripts (Liu et al, 2020) (*IL1B* [Liu et al, 2020] and *IL6* [Liu et al, 2020]), T17 cell–activating cytokine (*IL23A* [Liu et al, 2020]), and neutrophil chemoattractants (Metzemaekers et al, 2020) (*CXCL1*, *CXCL5*, *CXCL6*, and *CXCL8*), compared with psoriasis in which the pathogenic role of T17 cells and neutrophils is well-established (Kim and Krueger, 2017) (FCH > 2 and FDR < 0.05). The levels of *IL17A* and *IL17F* expression in late HS were higher than in lesional psoriasis, and surprisingly, the level of *IL17F* was also higher in early HS than in active psoriasis (Figure 6b and Supplementary Figure S4).

Finally, differentially expressed genes between early and late HS in the single-cell transcriptomic data confirmed those identified by spatial transcriptomics, including (i) increased expression in late HS of APC marker (*CD209* [Engering et al, 2002; Geijtenbeek et al, 2000]), MHC classes I and II molecules, plasma cell–related transcript (*JCHAIN* [Xu et al, 2020]), Igs, and cytotoxic T-cell transcripts (Bratke et al, 2005; Kim et al, 2021) (*GZMA* and *GZMK*) (FCH > 2 and FDR < 0.05) and (iii) higher expressions in early HS of AMP transcripts and *LCN2* (FCH < 0.5 and FDR < 0.05).

Taken together, these single-cell analyses from an independent cohort validate the spatial transcriptomic findings and highlight the potential pathogenic role of B cells in HS beyond antigen presentation and antibody production.

## DISCUSSION

The major challenge to diagnosis and treatment of HS comes from its evolving morphology and histology from superficial nodules in the early stage to deep dermal tunnels with nearby TLS and extensive fibrosis leading to scarring in the late stage (Dunstan et al, 2021; Nguyen et al, 2021; Sabat et al, 2020). Early-stage HS is often misdiagnosed as acne vulgaris/conglobata, leading to a diagnostic delay, leaving HS ignored and undertreated. On the other hand, late-stage HS with dermal tunnels and fibrosis do not resolve with medical treatments, and surgical management results in high recurrence and complications (Saylor et al, 2020).

The immune microenvironment in the HS skin has been studied in the context of interplays between late HS dermal structures: (i) keratinocytes in dermal tunnels expressing *IL17C* drives T17 cell activation and subsequent feed-forward inflammation (Navrazhina et al, 2021), (ii) TLSs near dermal tunnels support local T-cell and B-cell proliferation, (iii) B cells and plasma cells produce antibodies against keratinocytes, and (iv) fibroblasts promote B-cell aggregation by expressing *CXCL13* (Lowe et al, 2024; Yu et al, 2024). However, it has been unclear whether the T17 cell and B-cell inflammation sequentially precede or follow these late-stage structure formation.

We selectively collected skin specimens of early HS without clinical and histological evidence of dermal tunnels, TLS, or fibrosis and compared their spatial and single-cell transcriptomic profiles with those of late HS. Our study provided evidence that extensive immune activation, including a prominent B-cell signature, robust T17 cell pathway activation, and neutrophilic infiltration, is present in early HS before late HS structures are developed.

Early HS epithelium demonstrated an immune profile resembling psoriasis, marked by elevated expression of AMPs, stress keratins (*K6A*, *K16*, and *K17*), and *IL36G*, together with neutrophil chemokines (*CXCL1* and *CXCL6*) (FCH > 2 and FDR < 0.05) (Figures 2b and 4b). These epithelial alterations likely arise in response to T17 cell cytokines detected in the dermis, supporting the existence of a robust T17–keratinocyte axis (Nograles et al, 2008; Pfaff et al, 2017) and IL-17–chemokine–neutrophil axis (Flannigan et al, 2017; Hartupee et al, 2007; Wu et al, 2020) in early HS. Together, these findings provide a strong rationale for early intervention in HS using biologic agents that target the T17 cell axis, potentially halting disease progression. In addition, T17 cell cytokines (Liu et al, 2020) and receptor (*IL17F*, *IL21*, *IL22*, *IL23R*, *IL26*, and *TNF*), T17 cell–recruiting chemokine (*CCL20* [Harper et al, 2009]), and neutrophil-enriched transcript (*MPO* [Lin et al, 2024]) genes were more expressed in the deep dermis than in the superficial dermis in early HS (FCH > 2 and FDR < 0.05) (Figure 2c), whereas hair follicular epithelium in the superficial dermis did not express those inflammatory transcripts. Thus, although follicular occlusion may contribute to disease initiation, additional inflammatory mechanisms in the deep dermis of early HS may drive IL-17–chemokine–neutrophil axis beyond the hair follicle.

Another key finding in our study is the significant involvement of plasma cells and B cells in HS from its early stage. Consistent with previous studies (Gudjonsson et al, 2020; Lowe et al, 2020; Suh-Yun Joh et al, 2023), our spatial transcriptomic data demonstrated increased expression of plasma cell and B-cell lineage markers and Ig transcripts in early HS (Figures 2c), which was further supported by single-cell RNA sequencing showing expansion of plasma cell and B-cell populations (Figure 5c) and concordant differential gene expression patterns (Figure 6c). Notably, immunohistochemical staining confirmed the presence of CD20-positive B cells in early-stage HS lesions (Supplementary Figure S2). Together, these results suggest that early-stage HS already establishes an immune environment that facilitates B-cell maturation and plasma cell differentiation, potentially driving local antibody production and perpetuating chronic inflammation.

Interestingly, our study reveals that B cells in HS lesions could actively contribute to inflammation by expressing proinflammatory cytokines such as IL-16 (Figure 6a). IL-16 is a proinflammatory cytokine that recruits CD4^+^ T cells and modulates T-cell activation, which may further amplify T-cell–driven inflammation in HS (Center and Cruikshank, 1982; Mathy et al, 2000; Skundric et al, 2015). Although IL-16 is not classically regarded as a canonical B-cell–derived cytokine, prior studies have demonstrated that activated or neoplastic B cells can express and secrete IL-16 in chronic inflammatory or tumor-associated settings (Guan et al, 2025; Sharma et al, 2000; Skundric et al, 2006; Wen et al, 2025). Together, these observations raise the possibility that B cells in HS may acquire atypical cytokine programs and contribute to local inflammatory circuits beyond antigen presentation and antibody production. However, because these findings are based on transcriptional data, definitive assignment of IL-16 protein expression to B cells will require future validation using orthogonal approaches.

T-cell–derived *CXCL13* has been shown to recruit B cells and promote TLS formation in chronic inflammatory settings (Pitzalis et al, 2014; Rao et al, 2017). Prior studies have provided quantitative evidence of prominent B-cell infiltration and TLS formation in HS, particularly in advanced disease. Gudjonsson et al (2020) reported significant enrichment of plasma cells and B cells in HS compared with that in the controls, whereas Lowe et al (2020) identified increased B-cell infiltration in more severe (fibrotic end-stage) HS and demonstrated responsiveness to TNF-α blockade. More recently, Lowe et al (2024) showed that TLSs are present in a subset of advanced HS lesions, supporting ongoing B-cell maturation and class switching. Our spatial transcriptomic data further highlight an upregulation of the key B-cell chemoattractant *CXCL13* (Takagi et al, 2008) in the superficial dermis of early HS (Figures 2c and 4d), with single-cell transcriptomic data confirming that T cells are the major source of this chemokine (Figure 6a). In late-stage HS, prior studies have identified *CXCL13*-expressing inflammatory fibroblasts as a major cell population recruiting B cells and promoting the formation of TLSs (Lowe et al, 2024; Yu et al, 2024). Consistent with this stage-dependent distinction, our immunohistochemical analyses demonstrate CXCL13 protein expression within T-cell–rich inflammatory aggregates in early HS and a more diffuse, stromal-associated CXCL13 staining pattern in late HS (Supplementary Figure S5). Together, these observations suggest a stage-dependent shift in the spatial and cellular context of CXCL13 expression that may contribute to the transition from localized inflammation in early HS to the widespread inflammation in advanced disease.

Currently available therapies for moderate-to-severe HS include TNF-α inhibitor adalimumab, the IL-17A inhibitor secukinumab, and the IL-17A/F inhibitor bimekizumab. These therapies have been approved on the basis of the study of the inflammatory properties of mid-late-stage HS lesions, usually Hurley stages II and III. Although topical treatments have shown benefit in early-stage HS (Schell et al, 2025), no systemic therapies have been evaluated with the aim of preventing progression from early to late disease. We identified common pathways of immunological activation that are present in both early and late lesions of HS but tend to intensify as the disease progresses. Deep infiltrates of activated cells that we observed in early-stage lesions may not be sufficiently controlled with topical therapies and may therefore require the use of systemic anti-inflammatory drugs, as is now used in the treatment of later stages of the disease. Potentially, the interruption of B-cell activation or T17 cell pathways early in the course of disease might be able to prevent disease progression, which would need to be tested in future prospective trials.

A limitation of this study is related to the single-cell RNA-sequencing methodology used. Our single-cell dataset was generated using the “crawled-out” (migrating cell) approach, which is optimized for capturing viable immune cells but inherently underrepresents structural cells, particularly fibroblasts embedded within the extracellular matrix. As a result, direct quantitative comparisons of *CXCL13* expression between T cells and fibroblasts using single-cell RNA-sequencing data are limited. Therefore, to evaluate the proposed stage-dependent change in the cellular context of *CXCL13* expression, we relied primarily on immunohistochemical analyses, which preserve tissue architecture and avoid dissociation-related biases.

Another limitation of this study is that spatial transcriptomics captures transcripts at a spatial resolution encompassing multiple cells, restricting the ability to precisely resolve cell-type–specific gene expression, particularly in heterogeneous tissues as HS (Cheng et al, 2023). To address this, we validated key expression patterns using single-cell transcriptomics in an independent cohort. In addition, our interpretations of B-cell function by disease stage and the potential role of early infiltrating T cells in *CXCL13* production are based on transcriptional data and lack direct functional validation, warranting further study. Similarly, the presence of T-cell–associated transcriptional programs should be interpreted within the context of transcriptomic evidence and will require further functional validation.

Finally, our analyses compare early- and late-stage HS lesions from different individuals, and not all early lesions necessarily progress to late-stage disease with sinus tract formation. Not all early HS lesions evolve into later-stage disease, and clinical trajectories are variable; therefore, the early-versus-late differences identified in this study should be interpreted as cross-sectional contrasts rather than deterministic progression pathways. Future within-patient longitudinal studies will be required to directly characterize progression-associated changes.

Taken together, our results highlight the early and sustained activation of T17 cell pathway, coupled with B-cell enrichment extending into the deep dermis, emphasizing the aggressive nature of HS even in its initial stages. Future studies should explore the potential benefits of early T17 cell axis–targeting therapies and assess the efficacy of B-cell–directed treatments, such as CD20 inhibitors.

## MATERIALS AND METHODS

Skin biopsies were obtained from active lesions of 8 patients with early-stage HS (early HS) and 7 patients with late-stage HS (late HS). Classification was made at the biopsy site: early HS was defined by inflammatory nodules without dermal tunnels or fibrosis (Hurley stage 1), whereas late HS was defined by the presence of tunnels and scarring/fibrosis (Hurley stage 3) (Supplementary Figure S1). Patient characteristics are summarized in Supplementary Table S1.

Tissue specimens were collected with informed consent in accordance with the Declaration of Helsinki, and the study was approved by local ethics review boards. Comprehensive experimental methods and analyses are provided in supplementary materials (Supplementary Tables S2 and S3), including details of spatial transcriptomic experiments (region-of-interest selection, data integration, normalization, batch correction, and differential expression testing) and single-cell transcriptomic experiments with an independent patient cohort (data integration, normalization, clustering, and differential expression testing).

## Supplementary Material

Supplementary Table 1-3

1

Supplementary material is linked to the online version of the paper at www.jidonline.org, and at 10.1016/j.jid.2026.01.041.

## Data Availability

The spatial transcriptome data have been deposited in National Center for Biotechnology Information’s Gene Expression Omnibus and are publicly accessible through Gene Expression Omnibus Series accession number GSE294009. Gene Expression Omnibus sample accession number for each spatial transcriptome library is summarized in Supplementary Table S2. The single-cell transcriptome data have been deposited in National Center for Biotechnology Information’s Gene Expression Omnibus and are publicly accessible through Gene Expression Omnibus Series accession numbers GSE295706, GSE278330, GSE220116, and GSE151177. Gene Expression Omnibus Sample accession number for each single-cell library is summarized in Supplementary Table S3.

## Abbreviations:

AMP: antimicrobial peptide
FCH: fold change
FDR: false discovery rate
HS: hidradenitis suppurativa
MHC: major histocompatibility complex
T17: type 17 T
TLS: tertiary lymphoid structure
