# Supplementary material for: Spatial transcriptomics maps early B-cell and T-cell activation in hidradenitis suppurativa
Source: J Invest Dermatol. Author manuscript; Available in PMC 2026 Sep 8. (PMC13552446; doi:10.1016/j.jid.2026.01.041)
Supplement: Supplementary file 1 [file NIHMS2203138-supplement-1.pdf]

SUPPLEMENTARY MATERIALS AND METHODS

Human specimens

Hidradenitis suppurativa (HS) and acne conglobata (AC) skin specimens were collected at the Department of Dermatology, University of California, Davis (Sacramento, CA); Dermatology Section, Veterans Affairs Northern California Health Care System (Mather, CA); and the Department of Dermatology, Cutaneous Biology Research Institute, Yonsei University College of Medicine (Seoul, Republic of Korea). Skin biopsies were obtained from active lesions of 8 patients with early-stage HS (early HS) and 7 patients with late-stage HS (late HS). Classification was made at the biopsy site: early HS was defined by inflammatory nodules without dermal tunnels or fibrosis (Hurley stage 1), whereas late HS was defined by the presence of tunnels and scarring/fibrosis (Hurley stage 3) (Supplementary Figure S1). Patient characteristics are summarized in Supplementary Table S1.

All the human tissue specimens were collected with informed consent in accordance with the Declaration of Helsinki, and the study was approved by local ethics review boards. Patient demographics and skin biopsy locations are summarized in Supplementary Table S1.

Spatial transcriptomic experiments

We have created a spatial transcriptomic dataset of inflammatory skin diseases (HS, AC, psoriasis, and normal control) commonly using GeoMx Digital Spatial Profiling (DSP) technology (Nanostring Technologies) (Cho et al, 2025). The FASTQ-sequencing files, digital count conversion files, and associated metadata have been deposited in the National Center for Biotechnology Information (NCBI)'s Gene Expression Omnibus (GEO) (www.ncbi.nlm.nih.gov/geo) and are publicly accessible (GSE294009). HS and AC spatial transcriptomic data from the dataset were integrated with the investigators' published HS spatial transcriptomic data (Suh-Yun Joh et al, 2023) for the downstream comparison between early-stage HS and late-stage HS. Supplementary Table S2 summarizes spatial transcriptomic data analyzed for this study, including NCBI GEO sample accession number, disease classification, contributing institution, demographics, and spatial transcriptomic data information.

For the spatial transcriptomic experiments, tissue samples were sectioned and mounted on BOND Plus slides (Leica), following the manufacturer's guidelines. Formalin-fixed and paraffin-embedded tissue sections were deparaffinized using CitriSolv and rehydrated through a series of 100% ethanol, 95% ethanol, and 1X PBS. Antigen retrieval was performed using 1X Tris-EDTA (pH 9) at 100 °C for 15 minutes, followed by enzymatic digestion with 1 μg/ml proteinase K at 37 °C for 15 minutes. The tissues were then incubated with 10% neutral buffered formalin for 5 minutes, followed by 5 minutes with neutral buffered formalin stop buffer.

All tissue slides were incubated overnight at 37 °C with RNA hybridization probes using the GeoMx Whole Transcriptome Atlas Panel (18,677 genes). After overnight hybridization, slides were stained for 1 hour at room temperature with morphology markers in a humidity chamber: pan-cytokeratin (epithelial cell marker), CD45 (immune cell marker), and SYTO 13 (nuclear stain).

The stained slides were then loaded onto the GeoMx DSP instrument for spatial RNA profiling. Regions of interest (ROIs) were selected on the basis of scanned images loaded onto the DSP instrument. ROIs were designated into 4 distinct regions: epidermal epithelium, dermal epithelium, superficial dermal inflammatory aggregate, and deep dermal inflammatory aggregate. The epidermal epithelium was defined as the region above the dermis, whereas the dermal epithelium was designated as the area within the dermis. In early-stage HS, this included epithelium extending into the dermis, whereas in late-stage HS, it referred to epithelium forming sinus tracts. Superficial dermal inflammatory aggregate was defined as immune cell clusters adjacent to the epidermal epithelium, whereas deep dermal inflammatory aggregate was designated as immune cell populations located near the dermal epithelium or in deeper dermal regions.

The indexed oligonucleotides were then released under UV light and collected into a 96-well plate (DSP collection plate). The plate was dried on a PCR thermal cycler at 65 °C for 1 hour. Library construction was executed according to the NanoString GeoMx-NGS Read out Library Prep protocol. The PCR amplification utilized NanoString Seq Code primers, and the resulting products were combined in equivalent volumes before undergoing 2 rounds of AMPure XP bead purification (Beckman Coulter, catalog number A63880). Paired-end sequencing (2 × 151 bp) was performed using an Illumina NovaSeq 6000 system.

Spatial transcriptomic data integration, normalization, and batch correction

After transforming the sequenced FASTQ files into Digital Count Conversion format using the Nanostring GeoMx NGS Pipeline, we integrated and normalized the data and corrected batch effects using the standR package in R, version 4.2.2. Data normalization was performed using 'geomxNorm' function, followed by batch effect correction with RUV4 using the 'geomxBatchCorrection' function from the standR package (Liu et al, 2024). Because GeoMx DSP generates multiple ROIs per donor, which may introduce intradonor correlation, RUV4-based batch correction was used to capture latent sources of variation related to donor- and slide-level effects. This approach mitigates potential pseudoreplication arising from nonindependence of ROIs derived from the same biopsy while preserving spatial resolution.

The analysis was conducted with parameter k, which represents unwanted variation factors, set to 2. To understand how technical and biological factors influenced the data's variance structure, we performed principal component analyses on the quantile-normalized expression values after $\log_2(n+1)$ transformation. This revealed that the primary component corresponded to the aligned read-depth metric. To mitigate this technical effect, we employed the removeBatchEffect function from the Limma package to adjust the quantile-normalized expression values.

Spatial transcriptomic data differential expression testing

We performed differentially expressed gene (DEG) analysis by comparing each spatial location with the epidermal epithelium of AC. We selected the epidermal epithelium of AC as a control because AC manifests a common

pathophysiological feature with HS—follicular occlusion (Jastrząb et al, 2023)—yet its epidermal compartment is expected to exhibit lower levels of inflammation, allowing us to distinguish HS-specific transcriptomic changes.

Direct dermis-to-dermis comparisons with AC were not performed because the AC dermis is intrinsically paucicellular, and during quality-control and optimization steps, ROIs placed in the AC dermis frequently failed to meet the minimum nuclei count and sequencing read-depth thresholds required for robust normalization. Accordingly, DEG results derived from comparisons with AC epidermis should be interpreted as reflecting combined effects of immune cell infiltration and inflammatory activation rather than transcriptional changes within a matched dermal cell population.

We also conducted a DEG analysis comparing each location of early HS with the corresponding region in late HS.

DEG analysis was performed using the limma-voom framework in R (version 4.2.1). The spatial expression data were converted to a DGEList object, and a design matrix was constructed incorporating disease status and RUV (Remove Unwanted Variation) factors to account for technical variations. The biological coefficient of variation was estimated with robust estimation, followed by voom transformation to convert counts to $\log_2$ counts per million with associated precision weights. The transformed data were fitted to a linear model, and contrasts were established for pairwise comparisons between conditions. Empirical Bayes moderation was applied using eBayes with robust estimation to compute moderated *t*-statistics. Together, the limma-voom framework incorporating precision weighting, robust variance estimation, and empirical Bayes moderation stabilizes variance estimates across ROIs and limits inflation of statistical significance arising from unequal ROI representation across donors.

The analysis yielded results, including log-fold changes, average expression levels, and adjusted *P*-values (Benjamini−Hochberg correction). Results for each contrast were exported as separate files containing comprehensive information about DEGs between conditions.

Single-cell transcriptomic experiments

We have created a single-cell transcriptomic dataset of early-stage HS, late-stage HS, psoriasis lesional skin, and psoriasis nonlesional skin, commonly using droplet-based single-cell RNA-sequencing technology (10X Genomics). The single-cell RNA-sequencing data analyzed in this study were generated from an independent patient cohort and were not derived from the same patients with HS used for the spatial transcriptomic analyses. These data were used for cross-platform validation rather than direct patient-matched comparisons. The FASTQ-sequencing files, gene expression matrix files, and associated metadata have been deposited in the NCBI's GEO and are publicly accessible (GSE295706). The HS and psoriasis single-cell transcriptomic data were integrated with the investigators' published HS, psoriasis, and control single-cell transcriptomic data (GSE220116, GSE278330, and GSE151177) for the downstream comparison between early-stage HS and late-stage HS (Kim et al, 2025, 2023b, 2021). To minimize batch-to-batch variability, all the single-cell libraries were generated by 1 investigator under the same experiment protocol. Supplementary Table S3 summarizes single-cell transcriptomic data analyzed for this study, including NCBI GEO sample accession number, disease classification, contributing institution, demographics, and single-cell transcriptomic data information.

For the single-cell transcriptomic experiments, harvested skin biopsy tissues were placed in 0.2% Dispase II (Sigma-Aldrich) and incubated in a humidified incubator at 37 °C and 5% carbon dioxide for 3 hours. Then, the epidermis and dermis were separated with forceps and sliced into small pieces with #10 blade scalpels. The small pieces of epidermis and dermis were separately incubated in RPMI-1640 medium with L-glutamine (Cytiva) supplemented with 10% human albumin serum (Sigma-Aldrich) in a humidified incubator at 37 °C and 5% carbon dioxide. After 48 hours, cells that had emigrated from the epidermis and dermis were harvested and filtered through a 70-μm cell strainer (Corning) and a 40-μm cell strainer (Corning). Because this "crawled-out" (migrating cell) approach selectively captures cells that actively emigrate from skin explants, it is inherently enriched for viable immune cells and underrepresents structural cells such as keratinocytes and fibroblasts that are tightly embedded within the extracellular matrix. This approach is consistent with our previous human skin single-cell studies (Kim et al, 2025, 2023a, 2023b, 2021) and was equally applied to all the skin samples in this study.

Then, we prepared the single-cell suspension using the 10x Genomics Chromium Single Cell 3′ Reagents Kit (10X Genomics). The 10x Genomics Chromium Single Cell 3′ Reagent Kit chemistry version for each single-cell library is summarized in Supplementary Table S3. The appropriate volume of each sample was diluted to recover 5000−10,000 cells. Subsequently, the single-cell suspension, Gel Beads, and oil were added to the 10X Genomics single-cell chip. After droplet generation, the samples were transferred into PCR tubes, and we performed reverse transcription using a ProFlex PCR Thermocycler System (Applied Biosystems). After reverse transcription, cDNA was recovered using a recovery agent provided by 10X Genomics, followed by a silane DynaBeads clean-up as outlined in the user guide. The sequencer for each single-cell library is summarized in Supplementary Table S3. The raw sequencing data from single-cell libraries were processed using the 10X Genomics standard sequencing protocol. The FASTQ files were aligned to the human genome reference sequence GRCh38. Cell Ranger (10X Genomics) was applied to FASTQ files to generate files containing a barcode table, a gene table, and a gene expression matrix.

Single-cell transcriptomic data integration, normalization, and batch correction

After transforming the sequenced FASTQ files into gene expression matrix files using the 10X Genomics Cell Ranger pipeline, we used the Seurat package installed in R for the downstream single-cell RNA sequencing data analysis (Satija et al, 2015). For each single-cell library, genes expressed in <3 cells, cells expressing <100 or >5000 unique genes, and cells with a mitochondrial gene percentage of >25% were

filtered out to eliminate partial cells, doublets, and damaged cells. Ubiquitously expressed ribosomal protein-coding (RPS and RPL) and MALAT noncoding RNA, microRNA, and small nucleolar RNA genes were excluded from the analysis. Seurat objects were created, followed by normalizing data, scaling data, and finding variable 2000 genes. To mitigate batch effects introduced by different reagent kit versions and sequencers, we applied Seurat's canonical correlation analysis−based data integration framework, in which integration anchors (anchor cell pairs) were identified across datasets as mutual nearest-neighbor cells representing shared biological states, and these anchors were used to align datasets and correct batch effects (Stuart et al, 2019).

Single-cell transcriptomic data clustering analysis and nonlinear dimension reduction

Principal component analysis and graph-based clustering analysis were performed with the Seurat package installed in R. A total of 60 principal components were selected for the Uniform Manifold Approximation and Projection for Dimension Reduction. With a resolution of 0.4, the cells were clustered by the FindClusters function. When 5000−10,000 cells are submitted to a microfluidic platform for droplet-based single-cell library construction, 2.3−4.6% of single-cell data could be technical artifacts caused by cell doublets formed during cell capture (Chromium Next GEM Single Cell 3' Reagent Kits User Guide, 10X Genomics). To remove the clusters with possible doublets for downstream single-cell analysis, we excluded clusters expressing gene signatures of more than 2 different immune cell types. To compare cells in different conditions within clusters representing each type of skin immune cells, we merged adjacent clusters of common immune cell subsets. Single-cell transcriptomic map was annotated by expert manual cell annotation (Clarke et al, 2021).

For visualization purposes, all captured cell populations, including keratinocytes and fibroblasts, were retained in the Uniform Manifold Approximation and Projection representation to provide a global overview of the cellular landscape across conditions. Cell-type proportion analyses were calculated exclusively within the immune cell compartment. Structural cells, including keratinocytes and fibroblasts, were excluded from proportion calculations because their recovery is disproportionately affected by the migrating isolation method and does not accurately reflect their true abundance in tissue. This approach minimizes technical bias and enables more robust comparisons of immune cell composition across disease stages.

Single-cell transcriptomic data differential expression testing

DEGs between early-stage HS, late-stage HS, and psoriasis were tested by the Likelihood-ratio test for single-cell gene expression (McDavid et al, 2013) using the FindMarkers function in the Seurat package installed in R. We selected psoriasis as a control because psoriasis manifests a common pathophysiological feature with HS−type 17 T cell activation, and the direct comparison of type 17 T cells between HS and psoriasis elucidated different type 17 T-cell activation mechanism in our previous studies (Kim et al, 2023b).

Single-cell transcriptomic ligand−receptor interaction analysis

Ligand−receptor (L−R) interaction analysis was performed with the iTALK R package and its curated human L−R database. Interactions between immune and stromal cell populations in HS were compared with those in psoriasis skin, consistent with the differential expression testing. Putative L−R interactions were ranked on the basis of the combined differential expression of ligands in ligand-expressing (sender) cells and receptors in receptor-expressing (receiver) cells. Interactions were retained if both L−R pairs were upregulated, whereas self-loops (autocrine interactions) were excluded. Link arrow thickness and width were scaled to sender and receiver $\log_2$ fold change values, respectively.

Statistical analysis

To define the DEGs, we applied cut offs of fold change >2 or fold change <0.5 and false discovery rate−adjusted $P < .05$. We used the Benjamin−Hochberg procedure to adjust *P*-values to control the false discovery rate. For L−R interaction analysis, *P*-values were also adjusted for multiple testing using the Benjamini−Hochberg method, and the resulting adjusted *P*-values were used for downstream analyses. Only L−R interactions with adjusted $P < .05$ either from the ligand or receptor were considered statistically significant.

Data availability

The spatial transcriptome data have been deposited in NCBI's GEO and are publicly accessible through GEO Series accession number GSE294009. GEO Sample accession number for each spatial transcriptome library is summarized in Supplementary Table S2. The single-cell transcriptome data have been deposited in NCBI's GEO and are publicly accessible through GEO Series accession numbers GSE295706, GSE278330, GSE220116, and GSE151177. GEO Sample accession number for each single-cell library is summarized in Supplementary Table S3.

SUPPLEMENTARY REFERENCES

Cho C, Haddadi NS, Kidacki M, Woodard GA, Shakiba S, Yldz-Altay Ü, et al. Spatial transcriptomics in inflammatory skin diseases using GeoMx digital spatial profiling: a practical guide for applications in dermatology. JID Innov 2025;5:100317.

Clarke ZA, Andrews TS, Atif J, Pouyabahar D, Innes BT, MacParland SA, et al. Tutorial: guidelines for annotating single-cell transcriptomic maps using automated and manual methods. Nat Protoc 2021;16:2749−64.

Danaher P, Kim Y, Nelson B, Griswold M, Yang Z, Piazza E, et al. Advances in mixed cell deconvolution enable quantification of cell types in spatial transcriptomic data. Nat Commun 2022;13:385.

Jastrząb B, Szepietowski JC, Matusiak Ł. Hidradenitis suppurativa and follicular occlusion syndrome: where is the pathogenetic link? Clin Dermatol 2023;41:576−83.

Kim J, Lee J, Kim HJ, Kameyama N, Nazarian R, Der E, et al. Single-cell transcriptomics applied to emigrating cells from psoriasis elucidate pathogenic versus regulatory immune cell subsets. J Allergy Clin Immunol 2021;148:1281−92.

Kim J, Lee J, Lee J, Kim K, Li X, Zhou W, et al. Psoriasis harbors multiple pathogenic type 17 T-cell subsets: selective modulation by risankizumab. J Allergy Clin Immunol 2025;155:1898−912.

Kim J, Lee J, Li X, Kunjravia N, Rambhia D, Cueto I, et al. Multi-omics segregate different transcriptomic impacts of anti-IL-17A blockade on type 17 T-cells and regulatory immune cells in psoriasis skin. Front Immunol 2023a;14:1250504.

Kim J, Lee J, Li X, Lee HS, Kim K, Chaparala V, et al. Single-cell transcriptomics suggest distinct upstream drivers of IL-17A/F in hidradenitis versus psoriasis. J Allergy Clin Immunol 2023b;152:656–66.

Liu N, Bhuva DD, Mohamed A, Bokelund M, Kulasinghe A, Tan CW, et al. standR: spatial transcriptomic analysis for GeoMx DSP data. Nucleic Acids Res 2024;52:e2.

McDavid A, Finak G, Chattopadyay PK, Dominguez M, Lamoreaux L, Ma SS, et al. Data exploration, quality control and testing in single-cell qPCR-based gene expression experiments. Bioinformatics 2013;29:461–7.

Satija R, Farrell JA, Gennert D, Schier AF, Regev A. Spatial reconstruction of single-cell gene expression data. Nat Biotechnol 2015;33:495–502.

Stuart T, Butler A, Hoffman P, Hafemeister C, Papalexi E, Mauck WM 3rd, et al. Comprehensive integration of single-cell data. Cell 2019;177:1888–902.e21.

Suh-Yun Joh C, Jeong S, Lee C, Lee HJ, Lee JH, Choi HS, et al. Spatial transcriptomic profiling reveals the pathophysiology of early-stage hidradenitis suppurativa. Br J Dermatol 2023;189:643–5.

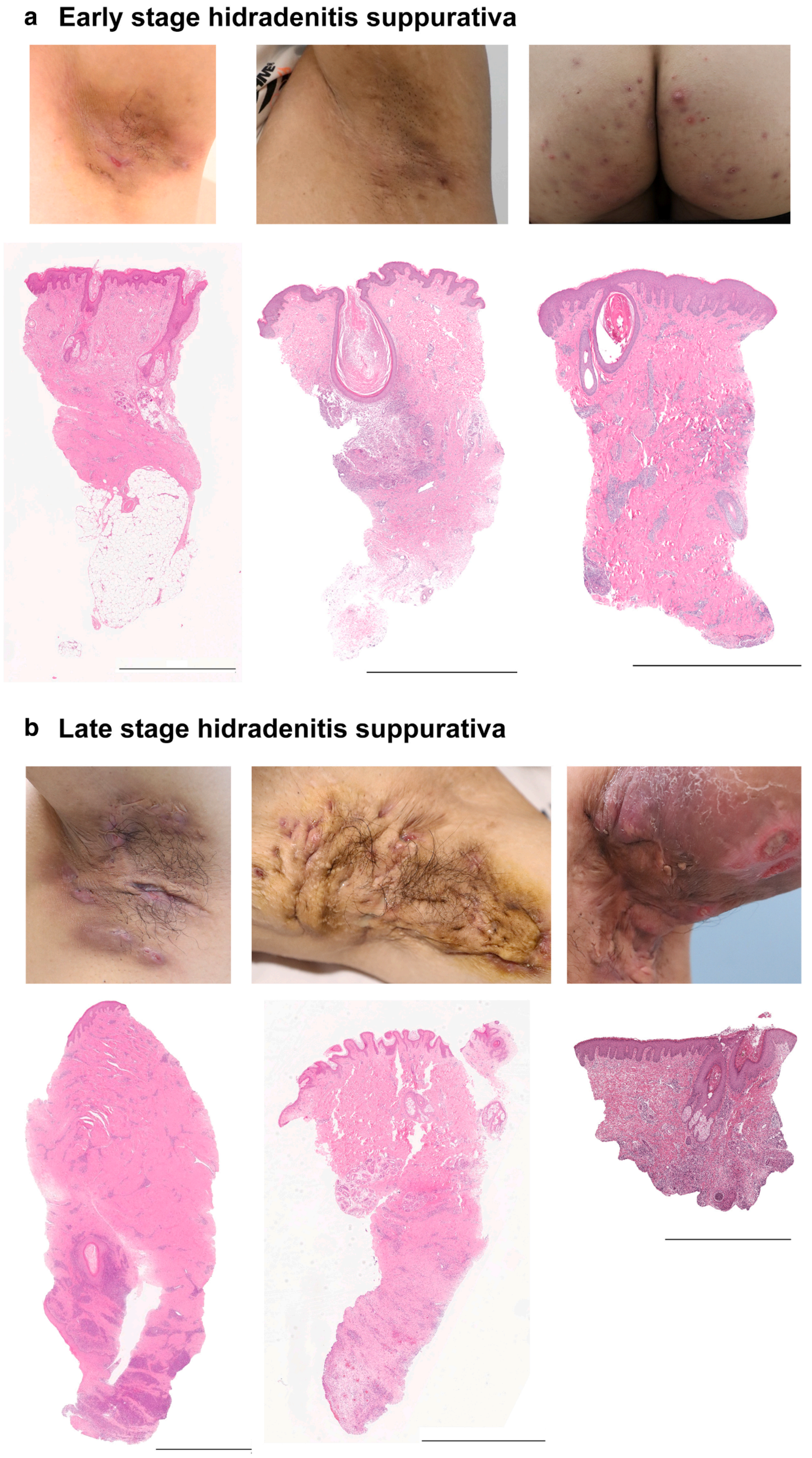

Supplementary Figure S1. Clinical and histological features of early- and late-stage hidradenitis suppurativa (HS). (**a**) Early-stage HS, shown with corresponding clinical photograph and H&E-stained tissue section. Bar = 2 mm. (**b**) Late-stage HS, presented with corresponding clinical photograph and H&E-stained tissue section. Bar = 2 mm. Written informed consent for publication of the clinical images was obtained from the subjects or their parent/guardian. HS, hidradenitis suppurativa.

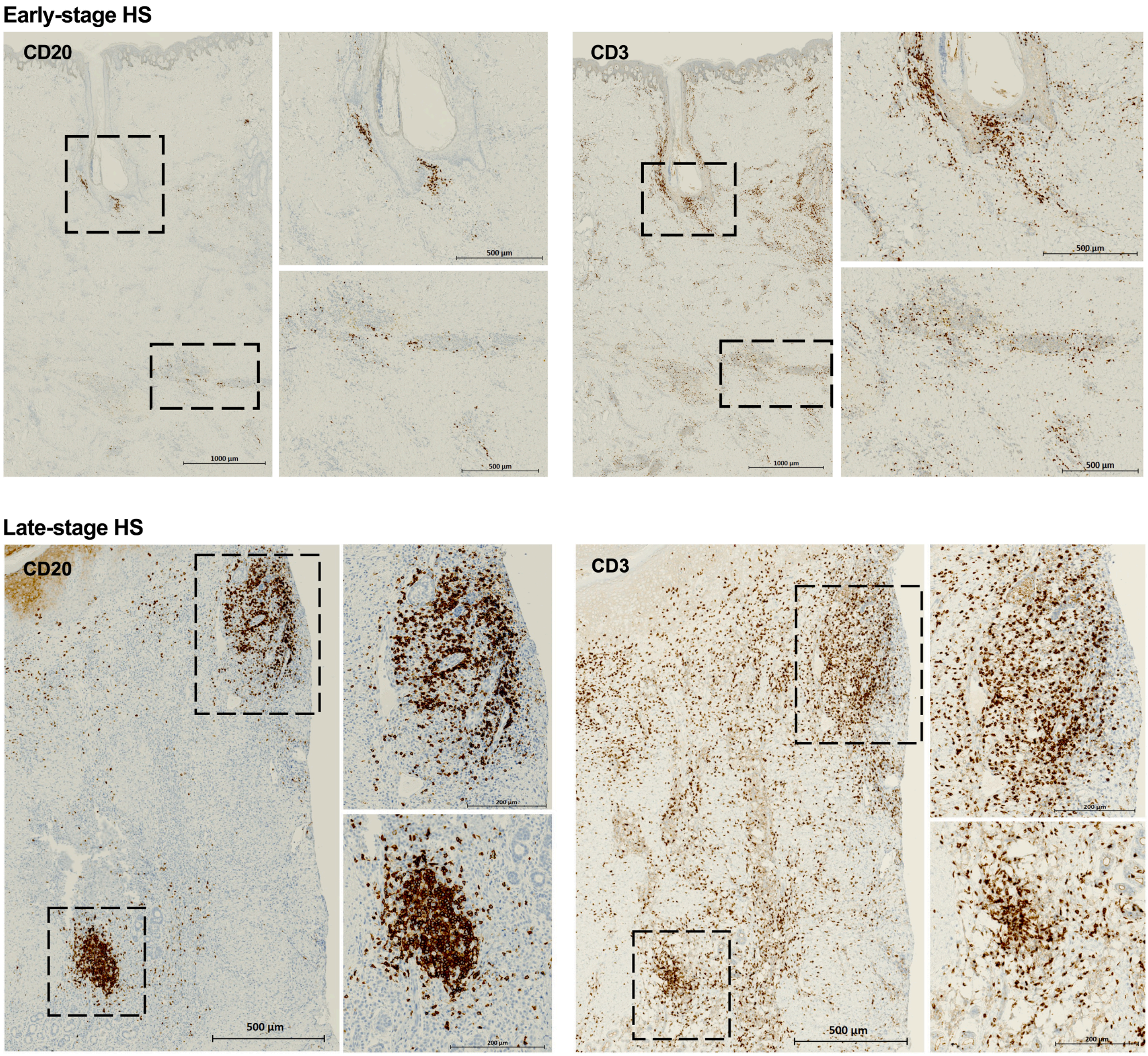

Supplementary Figure S2. Immunohistochemical staining of CD3 and CD20 in early- and late-stage HS. CD20-positive cells were detectable in early-stage HS lesions around dilated follicular opening and within the dermis, whereas CD3- and CD20-positive cells formed prominent aggregates consistent with TLS in late-stage HS. Bars = 1000, 500, and 200 µm in the corresponding insets. TLS, tertiary lymphoid structure.

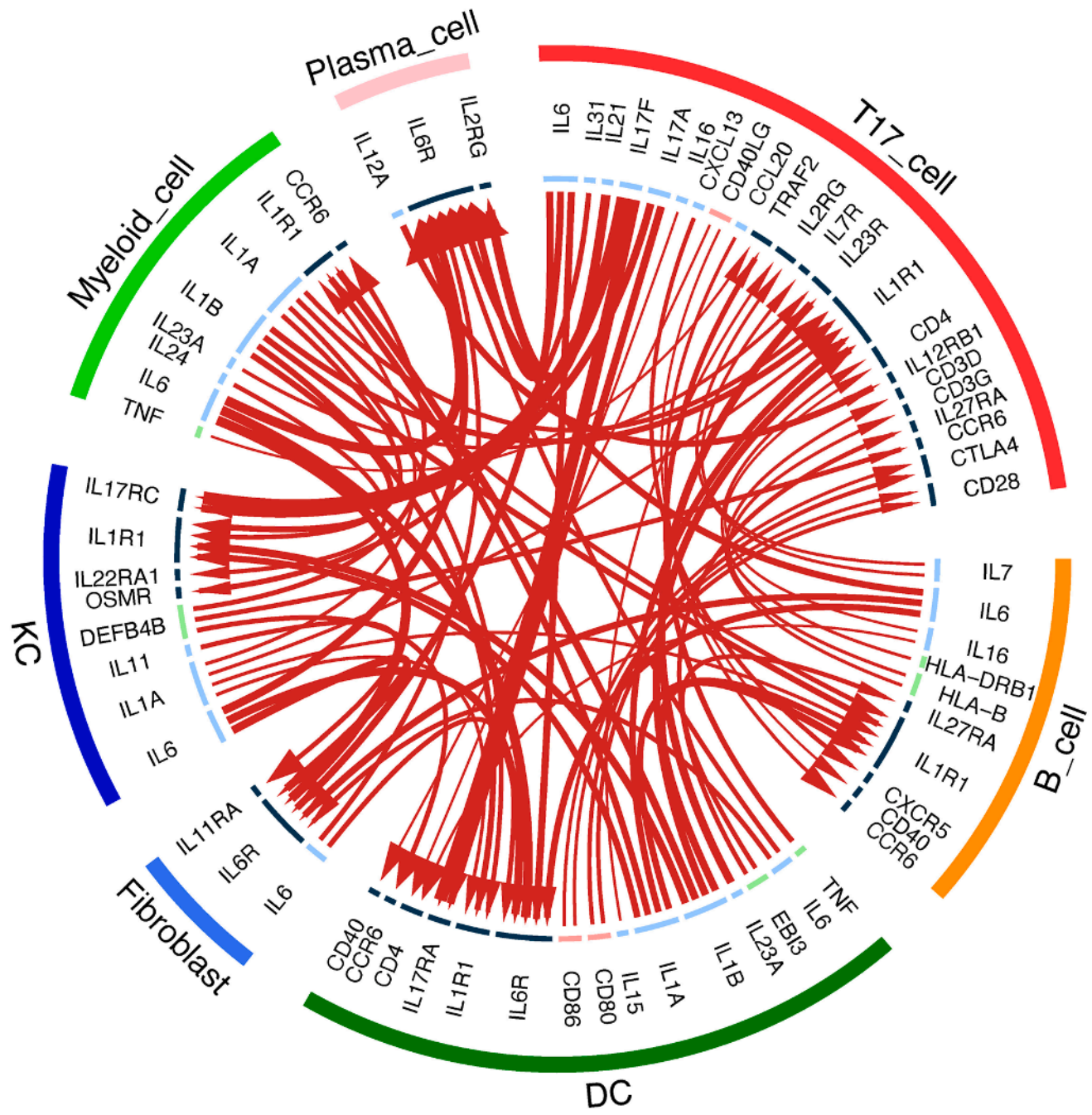

Supplementary Figure S3. L−R interaction analysis in HS. Chord diagram showing L−R interactions between immune and stromal cell populations in HS compared with those in psoriasis skin. Significant L−R interactions (adjusted $P < .05$) were observed between B cells and plasma cells with other immune subsets. Link arrow thickness and width are scaled to the $\log_2$ fold change values of the ligand (sender) and receptor (receiver), respectively. DC, dendritic cell; HS, hidradenitis suppurativa; L−R, ligand−receptor.

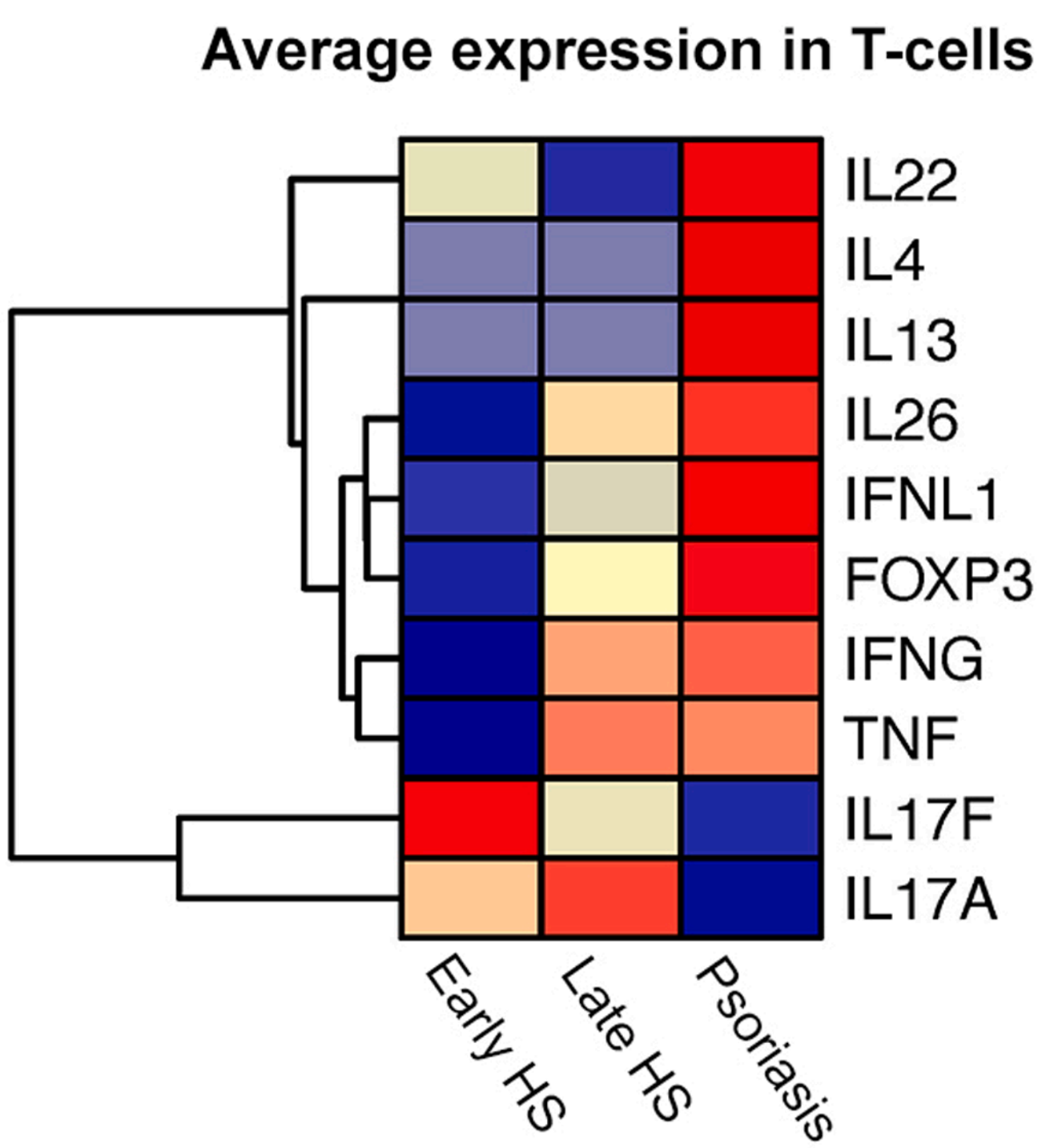

Supplementary Figure S4. Heatmap illustrating differential average gene expression in T cells from early-stage HS, late-stage HS, and psoriasis lesional skin. HS, hidradenitis suppurativa.

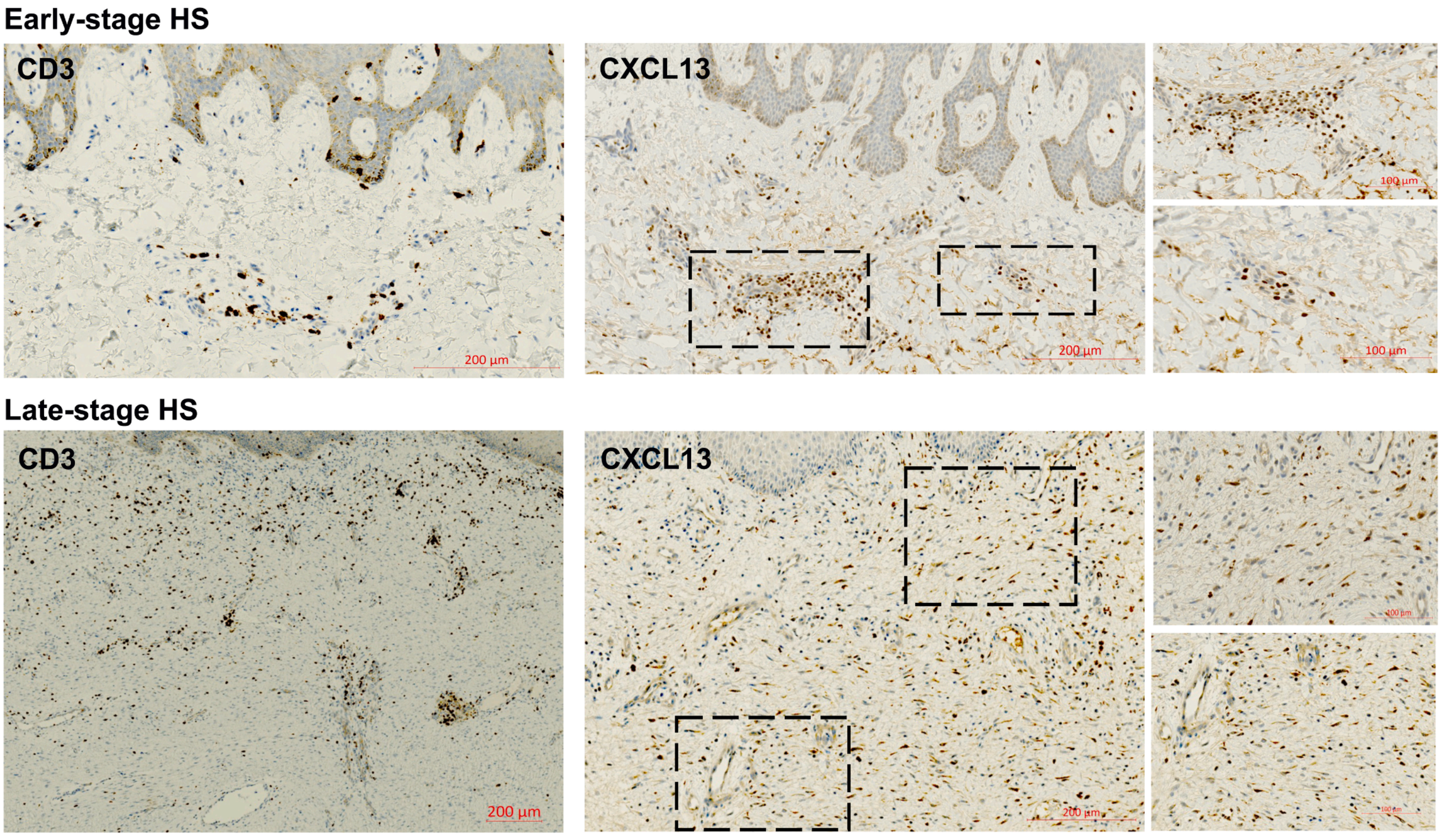

Supplementary Figure S5. Immunohistochemical staining of CD3 and CXCL13 in early- and late-stage HS using adjacent tissue sections. In early-stage HS, CXCL13-positive cells were observed within dermal inflammatory aggregates corresponding to CD3-positive T-cell–rich areas. In late-stage HS, CXCL13 staining was more diffusely distributed in the dermis with a wavy, fibrillar pattern, consistent with stromal cell–associated expression. Dashed boxes indicate regions shown at higher magnification. Bars = 200 and 100 µm. HS, hidradenitis suppurativa.
